# Supplementary material for: Effects of Trichoderma harzianum combined with Phanerochaete chrysosporium on lignin degradation and humification during chicken manure and rice husk composting
Source: Front Microbiol. 2025 Feb 28;16:1515931. doi: 10.3389/fmicb.2025.1515931 (PMC11906335; doi:10.3389/fmicb.2025.1515931)
Supplement: Supplementary file 1 [file Data_Sheet_1.zip › 6.Function/All/FunGuild/l1/heatmap_sample.pdf]

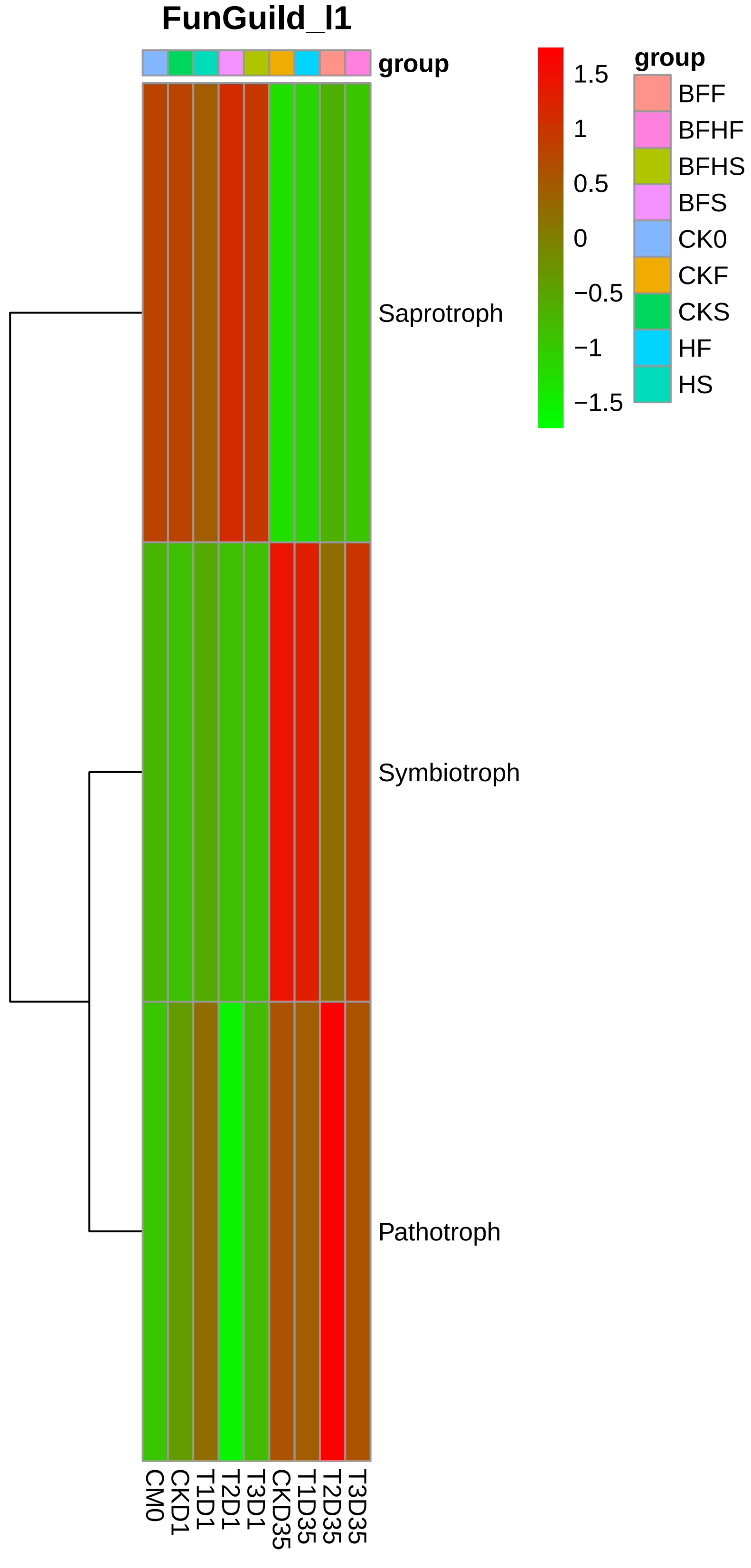
FunGuild_l1
group
Saprotroph
Symbiotroph
Pathotroph
CM0
CKD1
T1D1
T2D1
T3D1
CKD35
T1D35
T2D35
T3D35
1.5
1
0.5
0
−0.5
−1
−1.5
group
BFF
BFHF
BFHS
BFS
CK0
CKF
CKS
HF
HS
